# Tuberculosis-loop-mediated isothermal amplification implementation in Cameroon: Challenges, lessons learned and recommendations

**DOI:** 10.4102/ajlm.v11i1.1792

**Published:** 2022-08-26

**Authors:** Valerie F. Donkeng-Donfack, Suzanne M. Ongoulal, Yvonne J. Djieugoue, Yannick Kamdem Simo, Henri Manga, Danielle A.D. Tollo, Edwige M.A. Belinga, Vincent Mbassa, Jean L. Abena, Sara Eyangoh

**Affiliations:** 1 Mycobacteriology Unit, National Tuberculosis Reference Laboratory, Centre Pasteur du Cameroun, Yaoundé, Cameroon; 2 National Tuberculosis Control Program, Yaoundé, Cameroon; 3 Centre Pasteur du Cameroun, Yaoundé, Cameroon

**Keywords:** TB-LAMP, molecular test, implementation, roll out, notification

## Abstract

**Background:**

Until 2016, microscopy was the main tool for the early detection of pulmonary tuberculosis in Cameroon, especially in remote settings. Due to the poor sensitivity of microscopy, there was a need to implement a molecular assay in order to improve tuberculosis case detection.

**Intervention:**

In 2017, tuberculosis loop-mediated isothermal amplification (TB-LAMP), a molecular rapid diagnostic test recommended by the World Health Organization, was implemented in Cameroon as a replacement test of microscopy for initial diagnosis of pulmonary tuberculosis and also as a follow-on test to microscopy for smear-negative sputum specimens. A roll out plan for TB-LAMP implementation in Cameroon had been developed from January 2017 to April 2017, followed by initial implementation at four sites in May 2017. Additional sites were added progressively.

**Lessons learnt:**

The use of TB-LAMP as a follow-on test to microscopy for smear-negative sputum specimens helped in the detection of tuberculosis in 14.77% of those who were sputum-smear negative in 2019. Tuberculosis-loop-mediated isothermal amplification usage as an initial test, followed by testing with Xpert MTB/RIF for rapid tuberculosis and rifampicin resistance detection during tuberculosis mass screening campaigns, reduced the turn-around time by 73.23% as compared to when the Gene Xpert instrument was used alone.

**Recommendations:**

The implementation and scaling up of TB-LAMP in Cameroon contributed to increase access to tuberculosis molecular diagnosis in remote settings and as such improved tuberculosis case notification. However, to better enhance this notification and optimise the use of a TB-LAMP instrument, a suitable sample transport system is recommended.

## Background

Tuberculosis remains a major public health problem worldwide and tuberculosis detection cases remain a challenge and a priority for tuberculosis care and control.^1,2^ However, microscopy which is the main tool for the early detection of tuberculosis, especially in resource-limited countries with a higher burden of tuberculosis, has a sensitivity of about 50% – 60%.^3^ This sensitivity is even lower in children, people living with HIV and people with extrapulmonary tuberculosis.^4^ In addition, this sensitivity is lower compared to molecular tests such as tuberculosis loop-mediated isothermal amplification (TB-LAMP) and GeneXpert.^5^

Culture, which is the gold standard method for *Mycobacterium tuberculosis* complex diagnosis, is time consuming due to the slow growth rate of *M. tuberculosis* complex and requires the use of sophisticated instruments such as the biosafety cabinet which is expensive, requires a lot of training and has a high cost of maintenance.^6^ Accordingly, to rapidly detect tuberculosis cases as well as improve case detection including negative smear cases, the implementation of molecular World Health Organization (WHO)-recommended rapid diagnostic assays as tuberculosis initial diagnostic test is recommended to improve tuberculosis testing efficiency and increase tuberculosis bacteriologically confirmed cases.^7^

In 2012, the National Tuberculosis Control Program (NTCP) of Cameroon introduced Xpert^®^ MTB/RIF (Cepheid, Sunnyvale, California, United States), an automated semi-quantitative nested real-time polymerase chain reaction for the rapid and simultaneous detection of *M. tuberculosis* complex and rifampicin resistance.^8^ However, Xpert^®^ MTB/RIF equipment was placed at the regional laboratories and recommended for tuberculosis diagnosis in specific categories of presumptive tuberculosis cases including: children (0–5 years), people living with HIV, contact patients with multidrug- or rifampicin-resistant tuberculosis, prisoners, refugees, patients previously treated for tuberculosis or those with treatment failures. In 2016, TB-LAMP was endorsed by the WHO to be used as a replacement test for sputum-smear microscopy. The WHO recommended to use TB-LAMP as the initial diagnosis test for the detection of pulmonary tuberculosis (PTB) in adults who present signs and symptoms consistent with PTB, and also as an add-on test following negative smear microscopy, particularly when additional testing of smear-negative sputum specimens is necessary.^9^ Cameroon is a tuberculosis burden country^10^ with an estimated annual incidence of 103 new cases per 100 000 inhabitants in 2017. The expected number of new cases each year in Cameroon is 47 000. However, only around 50% of tuberculosis cases are reported annually, meaning that around 50% of tuberculosis cases are still missing.^11^ Accordingly, the Ministry of Public Health of Cameroon, through the NTCP, decided to implement TB-LAMP (Eiken Chemical, Tokyo, Japan) in 2017, with the goal to increase access to molecular diagnosis and ultimately improve tuberculosis case findings.^9^ Tuberculosis-LAMP is the only molecular test recommended by the WHO to be used in primary health care for the diagnosis of active tuberculosis.^12^ This test is a manual assay that does not need an air-conditioned room or sophisticated equipment and is not affected by short power intervals or variations. Up to 14 samples can be tested at once using TB-LAMP equipment, up to 70 tests can be performed in 8 h,^9^ and the results can be read with the naked eye under ultraviolet light.^9^ Implementation of the TB-LAMP assay requires minimal laboratory infrastructure and few biosafety requirements. The TB-LAMP test is a less expensive test,^13^ with high sensitivity compared to microscopy and a similar sensitivity and specificity to Xpert MTB/RIF.^5,14,15,16^ Before Cameroon, only countries from Asia, including the Philippines, Vietnam, Cambodia and Myanmar, performed pilot implementation of TB-LAMP with no big issues and they received project funding. Since the endorsement of TB-LAMP by the WHO, some African countries other than Cameroon, such as Nigeria, Zambia, Kenya, Uganda, Angola, Ivory Coast, Uganda, Gambia and Senegal, performed pilot implementation of TB-LAMP and they would be preparing for the extension phase. However, amongst African countries, Cameroon is the country where the implementation process started, in 2017. This paper describes the challenges, lessons learned and recommendations for TB-LAMP implementation in Cameroon.

## Description of the intervention

### Ethical considerations

The Ministry of Health through the National Tuberculosis Control Program conducted the implementation of TB-LAMP in Cameroon. As such, no ethical approval was required.

### Policy development

In 2014, with the support of the Foundation for Innovative New Diagnostics, Cameroon participated in the evaluation of TB-LAMP. The Centre Pasteur du Cameroun, through the National Reference Laboratory, conducted this evaluation at Jamot Hospital in Yaoundé. The results showed that TB-LAMP displayed higher sensitivity (82.6%; 95% confidence interval [CI]: 76.9–87.2) compared to smear microscopy with 53.6% (95% CI: 46.8–60.3). Tuberculosis-LAMP showed a specificity of 96.0% (95% CI: 93.2–97.7) and microscopy, 99.0% (95% CI: 97.1–99.7). Meanwhile, the sensitivity and specificity of TB‑LAMP were similar to GeneXpert^®^ (89.9%; 95% CI: 85.0–93.3 and 97.0%; 95% CI: 94.4–98.4, respectively).^16^ The overall results of this evaluation was used by the WHO to approve the TB-LAMP assay in 2016 as a follow-on and replacement test for sputum-smear microscopy in the diagnosis of PTB in adults with signs and symptoms consistent with tuberculosis.^9^ Therefore, the Ministry of Health, through the NTCP, reviewed the policy about to be implemented and decided to introduce this new molecular diagnostic technique in Cameroon.

### Evidence base

Tuberculosis-LAMP implementation in Cameroon started in May 2017 with a 2-month pilot phase. Four laboratories in two regions (Centre and South) were selected for this phase based on their workload. The Centre and the South regions represent, respectively, about 18.0% and 3.5% of the total population of Cameroon, with a tuberculosis prevalence of about 229/100 000 and 129/100 000 inhabitants in 2017. The Centre region is amongst the three regions with the highest number of tuberculosis case notifications in Cameroon. The four laboratories involved during this pilot phase include Jamot Hospital, Mbalmayo and Bafia District Hospital in the Centre region and Ebolowa Regional Hospital in the South region. These laboratories were selected because of their higher workload during the first semester of 2017, compared to other laboratories in their respective regions. In addition, theses laboratories were not far from the National Reference Laboratory that oversaw training and monthly monitoring during this pilot phase. Nine laboratory technicians were trained, and each laboratory was equipped with a TB-LAMP machine and an uninterruptible power supply. In these four laboratories, only the TB-LAMP assay was used for routine diagnosis of PTB in adults. Data obtained after two months of implementation (June and July 2017) at the four pilot sites were compared to the results obtained at the same sites over the same period of the previous year (June 2016 and July 2016), where only microscopy was used for initial PTB diagnosis. The compared data showed a 30.0% increase in positivity rate between 2016 and 2017. Table 1 shows the number of patients who accessed the tests from the four pilot sites during the months of June and July 2017 and the proportion of positive to negative cases. These results served as an evidence base to guide and reinforce Cameroon’s decision to go ahead with the scale up of TB-LAMP in the country.

**TABLE 1:** Number of patients tested with tuberculosis loop-mediated isothermal amplification as initial tuberculosis diagnosis test in June 2017 and July 2017 in four diagnostic and treatment centres in Cameroon.

TB-LAMP test results	Centre region			South region Ebolowa – Regional Hospital
	Jamot Hospital	Mbalmayo District Hospital	Bafia District Hospital	
Positive	217	23	48	38
Negative	587	70	57	86

**Total**	**804**	**93**	**105**	**124**

### Search for funds

In order to scale up the implementation of TB-LAMP in Cameroon, the results obtained during the pilot phase were used by the NTCP to address Cameroon’s 2018–2020 request for Global Fund (GF) support. In 2018, this request was validated by GF to support TB-LAMP implementation in Cameroon. In addition to the equipment and reagents, the GF support also covered training of laboratory technicians and supervisors of the TB-LAMP sites. However, the allocated funds had neither fees for HumaLoop T instrument installations nor fees to conduct related activities such as meetings with clinicians. Furthermore, these funds did not consider additional materials not provided by the kits, including scissors, gloves, marker pens and tissue paper. Fortunately, these additional fees were covered by the government through the Tuberculosis National Reference Laboratory (TB-NRL) hosted at Centre Pasteur du Cameroun for an optimal implementation of the new technique. In addition, the government also supported sample transportation.

### Selection of settings

Twenty-one tuberculosis Diagnostic and Treatment Centers (DTCs) out of the 260 DTCs present in the country were selected in the 10 regions for the scaling up of TB-LAMP. Many reasons guided the selection of these DTCs. Nineteen of the DTCs are at remote settings and had only microscopy as a tuberculosis diagnostic tool. Two of the DTCs are tuberculosis regional reference laboratories. The workload of the selected DTCs was higher compared to the others. All 21 DTCs have electricity and easy access for sample transportation. This selection of sites for TB-LAMP extension was performed prior to the training of staff.

### Procurement, revision of tuberculosis national guidelines and tuberculosis diagnosis algorithms

The procurement of reagents and equipment (HumaLoop T and Uninterruptible *Power* Supply) was done through the Global Drugs Facility (GDF). During the procurement period, the NTCP revised the tuberculosis national guidelines and tuberculosis diagnosis algorithms in May 2018 to include TB-LAMP assay. On the revised tuberculosis national guidelines, the NTCP recommended the use of TB-LAMP at the DTCs where it was implemented as a replacement test for sputum-smear microscopy for initial diagnosis of PTB in adults with signs and symptoms consistent with tuberculosis. However, at the microscopy testing centres, TB-LAMP was recommended to be used as a follow-on test to smear microscopy for smear-negative sputum specimens in adults with signs and symptoms consistent with PTB.

### Steps for tuberculosis-loop-mediated isothermal amplification scaling up in Cameroon

The scaling up of TB-LAMP in Cameroon was performed in four steps including trainings, equipment installation, stakeholder engagement and sample transportation.

#### Training

**FIGURE 1:**
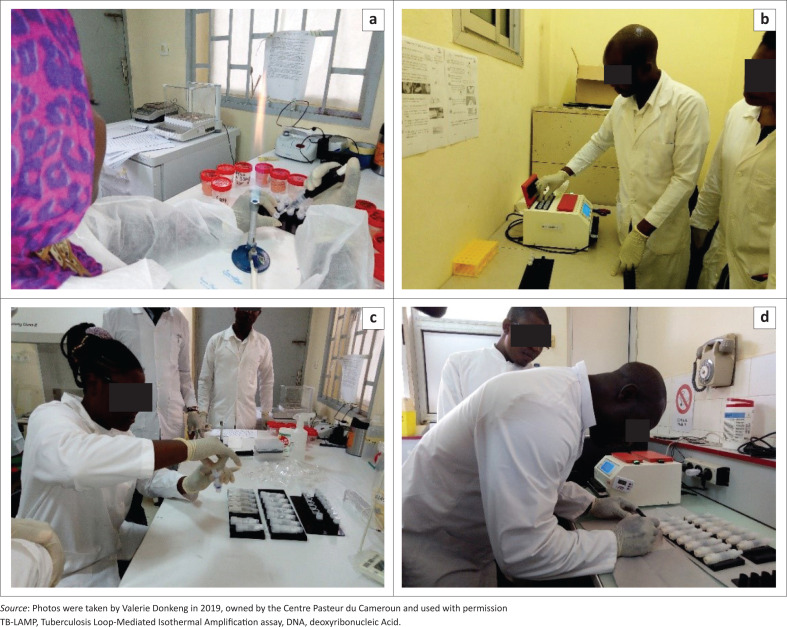
Tuberculosis loop-mediated isothermal amplification trainings during scaling up in Cameroon, June 2019 and August 2019. (a) Transfer of sputum sample to heating tube. (b) Removal of heating tube from the heating block of the HumaLoop T instrument. (c) DNA extraction. (d) Labelling of reaction tubes. Douala TB Regional Reference Laboratory 08-08-2019 (a, b) and Garoua TB Regional Reference Laboratory 20-06-2019 (c, d).

Trainings started with the training of trainers organised by the TB-NRL at the Centre Pasteur du Cameroun. Five trainers, including three from the TB-NRL and two from the pilot sites (Jamot Hospital and Ebolowa Regional Hospital), were trained over five days. They were then involved in the training of about 50 laboratory technicians. Trainings included theoretical and practical sessions (Figure 1). The objective of these trainings was to improve the technical capacities of laboratory technicians for better diagnosis of tuberculosis in the laboratory using HumaLoop T equipment. Due to budget constraints, only four sessions of three days each were organised for the training in the country. A minimum of 12 personnel were trained per session by two trainers with one HumaLoop T instrument. The training was laborious for the trainers because of the high number of trainees per session.

#### Installation of HumaLoop T instrument at selected sites

After training sessions, HumaLoop T instruments with uninterruptible power supplies were installed at the 21 selected sites. The role of the uninterruptible power supply is to allow the equipment to keep running for at least a period when the primary power source is lost. The installations were performed in the week following the training to enable technicians to quickly start TB-LAMP testing in their respective settings, with a fresh memory of the training. The activity of HumaLoop T instruments installation was not funded by the GF. The government covered this activity through the TB-NRL.

#### Stakeholder engagement

In order to ensure a successful implementation of TB-LAMP in Cameroon, the Ministry of Health, through the NTCP, had organised a sensitisation meeting in March 2018, on World TB Day, on the role of TB-LAMP in improving tuberculosis diagnosis in Cameroon. The meeting was chaired by the Minister of Health, together with the head of the NTCP and the General Manager of the Centre Pasteur du Cameroun. This meeting involved community leaders from high burden communities, tuberculosis active case finders, physicians, laboratory technicians, directors of TB-DTCs, the Ministry of Health, other stakeholders, and both national and international media. In addition to this meeting, regional sensitisation meetings were also organised. Attendees included physicians, nurses, bikers involved in sample transportation and other health staff involved in tuberculosis patient care. During the meetings, participants were informed about the introduction of the new tuberculosis diagnosis assay at the DTC and regional level, as well the role and the advantages of the new technology. Also, TB-LAMP algorithms were presented and discussed. The government funded these national and regional sensitisation meetings through the TB-NRL.

#### Sample transportation

The sample transportation system of smear-negative sputum specimens from microscopy centres to TB-LAMP centres for further testing was organised in all regions in 2019 in order to give all PTB presumptive cases the benefit of the TB-LAMP assay. According to the number of TB-LAMP laboratories in a region, microscopy laboratories were organised into pools, and with a TB-LAMP laboratory. This activity of smear-negative sputum specimen transportation was funded by the government. Bikers and public transport were used for transportation. City transportation used bikers whereas intercity transportation used intercity public transportation. Furthermore, laboratories were equipped with phones and communication credits to ensure communication between them and the bikers for sample collections and results deliveries, to reduce the turn-around time. The government funded this activity. The challenges were insufficient funds as well as the small number of bikers.

### Challenges

The implementation of TB-LAMP in Cameroon took two years (from May 2017 to May 2019). During these two years, 27 DTCs were gradually equipped with a HumaLoop T instrument. The implementation started with four machines in 2017, followed by two machines in 2018 and 21 machines in 2019. The government funded the first six machines and the GF the other 21. From the four pilot sites in 2017, the NTCP established a 3-year extension plan (2018–2020), supported by the GF. Many challenges accompanied this implementation.

#### Make pipette-60 set material available on the Global Drugs Facility catalogue

The procurement of reagents and equipment (HumaLoop T and Uninterruptible *Power* Supply) through the GDF took about 10 months between placement of order and delivery. The issue was due to the absence of the pipette set on the GDF catalogue. Therefore, Human and Eiken companies negotiated with the GDF to include pipette-60 set material on the GDF catalogue, to facilitate procurement.

#### Getting additional funds for tuberculosis-loop-mediated isothermal amplification implementation-related activities and for additional materials

For an efficient implementation of TB-LAMP, we requested additional funds from the government to conduct related activities, such as meetings with clinicians, and to purchase supplementary materials such as scissors, gloves, marker pens and tissue paper needed for TB-LAMP assays, but not provided with the kits.

#### Trainings of laboratory technicians

Organising a 3-day training session for a minimum of 12 technicians with only one HumaLoop T instrument was very challenging because of the reduced number of days and the high number of trainees. Accordingly, the TB-NRL proposed to the NTCP to reduce the number of trainees per session for the next year and to train technicians directly in their laboratory.

#### Increasing tuberculosis case notification

To increase tuberculosis case notification using a HumaLoop T instrument, the NTCP implemented the use of TB-LAMP in 2019 as a follow-on test to smear microscopy for smear-negative sputum specimens in adults with signs and symptoms consistent with PTB. This activity was done through an organised sample transportation system for transportation of smear-negative sputum specimens to TB-LAMP sites. This activity helped to detect up to 388 (14.77%) tuberculosis patients out of 3061 smear-negative sputum specimens transferred to TB-LAMP sites. These patients could have been released into the community and, as such, could have continued to spread the disease. In addition, the NTCP organised tuberculosis mass screening campaigns at 34 prisons in 2019 and the TB-LAMP assay was used as initial tuberculosis diagnosis followed by testing with Xpert MTB/RIF for tuberculosis and rifampicin resistance detection. Tuberculosis mass screening campaigns helped in the detection of 123 tuberculosis cases out of 3672 presumptive tuberculosis cases at the 34 prisons, and three rifampicin resistance cases. Table 2 presents the summary stats, by year, for the four initial sites and the number of positive cases picked up by TB-LAMP.

**TABLE 2:** Summary statistics for the four initial sites by year and number of positive cases picked up by tuberculosis loop-mediated isothermal amplification, Cameroon, 2017–2019.

Years	Sites			
	Jamot Hospital	Mbalmayo District Hospital	Bafia District Hospital	Ebolowa Regional Hospital
**2017**				
Positive	708	101	125	130
Negative	2307	401	318	627
Total	3015	502	443	757
Positivity rate (%)	23.48	20.11	28.21	17.17
**2018**				
Positive	1711	185	293	301
Negative	5570	859	933	1313
Total	7281	1044	1255	1614
Positivity rate (%)	23.49	17.72	23.34	18.64
**2019**				
Positive	1498	129	178	230
Negative	5546	627	620	1343
Total	7044	756	798	1573
Positivity rate (%)	21.26	17.06	22.3	14.62

## Lessons learnt

The implementation of TB-LAMP in Cameroon offered an opportunity for us to draw several lessons for an effective implementation. These lessons learned will be useful for countries that will start with TB-LAMP implementation after us.

### Pulmonary tuberculosis-loop-mediated isothermal amplification materials and its use

The TB-LAMP assay needs additional materials such as scissors, gloves, marker pens and tissue paper, that are not provided with the kits. Moreover, TB-LAMP assays required a positive and a negative control for each run. These controls provided by the kits include one tube with 0.4 mL of positive control and three tubes of 0.5 mL of negative control. The available volume of positive control is necessary for 12 runs of six samples/run. However, when a laboratory performs less than six samples/run, they will perform more than 12 runs and the 0.4 mL of positive control will not be sufficient. Also, using less than six samples/run leads to the consumption of more extraction and detection kits for controls. This situation could therefore lead to a stock shortage if the estimations did not consider it.

### Education of tuberculosis-loop-mediated isothermal amplification users

The use of TB-LAMP does not require a background in molecular biology. During TB-LAMP implementation in Cameroon, most of the laboratory technicians trained at the remote setting on the use of TB-LAMP for tuberculosis diagnosis were the ones performing microscopy with no background in molecular biology.

### Infrastructure

Tuberculosis-LAMP has been implemented in the laboratories performing microscopy as a routine test for tuberculosis diagnosis. Therefore, the same infrastructure used for microscopy was applied. There was no need to adjust or to modify infrastructure.

### Reduction of turn-around time during tuberculosis mass screening campaigns

In 2019, a diagnostic algorithm based on an initial testing with TB-LAMP, followed by testing with Xpert MTB/RIF to diagnose tuberculosis, reduced the turn-around time by 73.23% during mass campaigns in 34 prisons compared to Xpert MTB/RIF when used alone.

## Recommendations

### Search for funding

Implementation of TB-LAMP requires consideration, during forecasting, fees of related activities such as meetings and sample transportation, as well as additional materials not provided by the kits.

### Training

Trainings should include a maximum of four trainees for a 3-day session with one HumaLoop T instrument, and sufficient financial resources should be allocated for these training activities, so as to train technicians in their various laboratories with the use of their instrument.

### Pulmonary tuberculosis-loop-mediated isothermal amplification settings

The robustness of the HumaLoop T instrument makes it good for peripheral laboratories receiving large numbers of samples. This will avoid the use of less than six samples/run. With the help of solar panels, a HumaLoop T instrument can be used for tuberculosis active case finding in communities where there is no electricity.

### Implementation of a diagnostic algorithm based on an initial testing with tuberculosis-loop-mediated isothermal amplification followed by testing with Xpert MTB/RIF to diagnose tuberculosis

This approach improved early and rapid tuberculosis detection with an added advantage of providing rifampicin resistance status.

### Data management

DataToCare, a simple, customisable, and patient-centric application developed by Savics SRL (Brussels, Belgium), could help to record patient, physician and laboratory operator information, send results directly to the physician via SMS or email, and easily generate weekly, quarterly and annual reports.

### Conclusion

Despite some difficulties, the implementation of TB-LAMP in Cameroon was well conducted, and the TB-LAMP test is used as a WHO-recommended initial rapid diagnostic test for all people with signs and symptoms of tuberculosis. The challenges, lessons learned and recommendations resulting from this implementation will help other countries to have a more efficient implementation.
